# The synergy factor: a statistic to measure interactions in complex diseases

**DOI:** 10.1186/1756-0500-2-105

**Published:** 2009-06-15

**Authors:** Mario Cortina-Borja, A David Smith, Onofre Combarros, Donald J Lehmann

**Affiliations:** 1 Centre for Paediatric Epidemiology and Biostatistics, Institute of Child Health, University College London, 30 Guilford Street, London, WC1N 1EH, UK; 2 Oxford Project to Investigate Memory and Ageing (OPTIMA), Department of Physiology, Anatomy and Genetics, South Parks Road, Oxford, OX1 3QX, UK; 3 Neurology Service and Centro de Investigación Biomédica en Red sobre Enfermedades Neurodegenerativas (CIBERNED), Sevilla, Spain; 4 Marqués de Valdecilla University Hospital (University of Cantabria), 39008 Santander, Spain

## Abstract

**Background:**

One challenge in understanding complex diseases lies in revealing the interactions between susceptibility factors, such as genetic polymorphisms and environmental exposures. There is thus a need to examine such interactions explicitly. A corollary is the need for an accessible method of measuring both the size and the significance of interactions, which can be used by non-statisticians and with summarised, e.g. published data. The lack of such a readily available method has contributed to confusion in the field.

**Findings:**

The synergy factor (*SF*) allows assessment of binary interactions in case-control studies. In this paper we describe its properties and its novel characteristics, e.g. in calculating the power to detect a synergistic effect and in its application to meta-analyses. We illustrate these functions with real examples in Alzheimer's disease, e.g. a meta-analysis of the potential interaction between a *BACE1 *polymorphism and *APOE*4: *SF *= 2.5, 95% confidence interval: 1.5–4.2; *p *= 0.0001.

**Conclusion:**

Synergy factors are easy to use and clear to interpret. Calculations may be performed through the Excel programmes provided within this article. Unlike logistic regression analysis, the method can be applied to datasets of any size, however small. It can be applied to primary or summarised data, e.g. published data. It can be used with any type of susceptibility factor, provided the data are dichotomised. Novel features include power estimation and meta-analysis.

## Background

### The need

The remarkable progress made in the understanding of single-cause diseases has not yet been matched in the study of complex conditions. One problem is that susceptibility factors, e.g. genetic and environmental, all contribute risk that is to varying extents contingent on the presence of other factors [1-4]. Complex diseases cannot therefore be simply seen as due to the accumulation of many small independent effects. Rather, their very complexity lies in the interactions between contingent effects. Important effects may thus be missed if only single factors are independently examined (Discussion). The study of interactions between risk factors is thus central to the study of complex diseases.

Yet, unravelling interactions has proved confusing (Discussion). There is a need for a readily accessible method of measuring their strength, available to non-statisticians and applicable to summarised data and to datasets of any size. Methods are also needed to calculate the power to detect an interaction and to perform meta-analyses of interactions from published data; these two functions have not so far been readily available. There is a particular need for an accessible method for referees; untested claims of synergy are regularly published. Here we present a statistic, the synergy factor (*SF*), derived from logistic regression models, which aims to address these needs.

### Modelling interactions in case-control studies

This paper is about statistical interactions; thus, drawing inferences about biological causality is beyond its scope. In general, a statistical interaction arises "when the effect of one explanatory variable depends on the particular level or value of another explanatory variable" [5]. Interactions may correspond to deviations from additive or multiplicative models for the joint effects of two risk factors. This has been thoroughly explored by Berrington de González and Cox [6,7], with two procedures, one for each model.

Some epidemiologists, e.g. Rothman and Greenland [8], argue that assessment of interaction should be based on additive rate or risk models. These models are the norm in cohort studies. However, to assess interaction as departure from additive risks in case-control studies, three surrogate measurements of interaction based on the parameters of logistic regression models have been proposed [9,10]: the relative excess risk due to interaction, the attributable proportion due to interaction and the synergy index. Skrondal has shown [11] that only the synergy index may be validly used for this purpose and only after fitting a linear odds model.

In case-control studies, the parameter which is both estimable and interpretable as a relative risk is the odds ratio (*OR*) [11]. In such studies, the predicted joint effect of two genetic or other factors may be defined as the product of the effects of each factor alone. We therefore propose a single statistic, the synergy factor (*SF*), which depends on a multiplicative definition of the null hypothesis.

## Methods

A full description of the methodology for significance tests based on the *SF *appears in Additional file 1. We show there that ln(*SF*) is equivalent to the interaction term defined by two binary factors in a logistic regression model. We test the hypothesis of no interaction, using a Normal approximation for the statistic ln(*SF*)/stderr(ln(*SF*)), where the standard error of ln(*SF*) is easily obtained via the delta method [12]. This approximation is adequate even for relatively small sample sizes. We discuss a modification of the *SF *to cope with empty cells and propose two bootstrap approximations and a Bayesian inferential procedure that can be used as alternatives to the Normal approximation. We also propose methodology to calculate the power of significance tests and to perform meta-analyses based on the *SF*.

## Results

### The synergy factor (SF)

Let us assume we wish to estimate from a case-control study whether there is an interaction between any two (binary) factors, *x*_1 _and *x*_2_, in the risk of a certain (binary) condition. Taking subjects with neither factor as reference, we first estimate the *OR*s for factor *x*_1 _alone (*OR*_1_), factor *x*_2 _alone (*OR*_2_) and both factors combined (*OR*_12_). The *SF *is then defined as: *SF *= *OR*_12_/(*OR*_1 _× *OR*_2_) and is the ratio of the observed *OR *for both factors combined, to the predicted *OR *assuming independent effects of each factor. Susceptibility factors may be associated with increased or reduced risk, i.e. risk or protective factors, respectively (we make no assumptions about causality). In either case, interactions may be positive (synergy) or negative (antagonism). Thus, if *SF *> 1 (< 1), then there is a positive (negative) interaction between two risk factors. The opposite applies to protective factors.

To obtain the statistical significance of *SF*, construct a 4 × 2 table of the numbers of cases and controls in each of the 4 possible combinations of the two factors (e.g. Table 1). Then if *n*_1_, *n*_2_....*n*_8 _are the values of the 8 cells, application of the delta method [12] yields an asymptotic normal approximation to the standard error of ln(*SF*) as: . Since the null value is 0, the statistic *Z *= ln(*SF*)/stderr(ln(*SF*)) has asymptotically a standard normal distribution under the null hypothesis of no interaction.

**Table 1:** Odds ratios of Alzheimer's disease, taking subjects with the *BACE1 *rs638405 C allele and without *APOE*4 as reference

***BACE1***	***APOE*4**	**Controls**	**Cases**	***OR***
C+	-	125	80	Reference
GG	-	80	38	0.742
C+	+	48	74	2.409
GG	+	19	60	4.934

Totals		272	252	

Data from Nowotny et al 2001 [13].*APOE*4 = the ε4 allele of apolipoprotein E; *BACE1 *= the β-site APP-cleaving enzyme; C+ and GG refer to *BACE1 *exon 5 C/G (rs638405) genotypes; *APOE*4+ and *BACE1 *C+ pool homozygotes and heterozygotes of the respective alleles; *OR *= odds ratio

### Synergy between risk factors

Let us take the potential interaction in risk of Alzheimer's disease (AD) between the ε4 allele of apolipoprotein E (*APOE*4) and the GG genotype of the C/G polymorphism (rs638405) in exon 5 of the β-site APP-cleaving enzyme (*BACE1*) [13] (Table 1). Taking subjects with neither *BACE1 *GG nor *APOE*4 as reference, the *OR *for *BACE1 *GG alone was 0.742 and that for *APOE*4 alone was 2.409. That gave a predicted *OR *of 1.788 (= 0.742 × 2.409) for the combination, compared with an observed *OR *of 4.934. Hence: *SF *= 2.76 (= 4.934/1.788), 95% confidence interval (CI): 1.25–6.09, ln(*SF*) = 1.015, stderr(ln(*SF*)) = 0.404, *Z *= 2.25 and *p *= 0.012. Thus the null hypothesis of no interaction was rejected and significant synergy was found. The observed joint effect of the two variants was nearly three times greater than the predicted joint effect.

**Figure 1:**
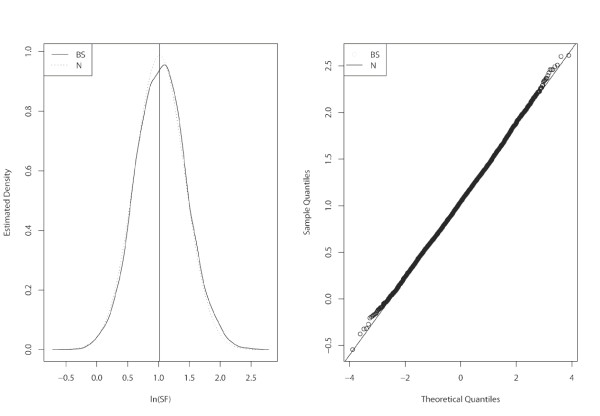
**Normal (N) and bootstrap (BS) approximations to the null distribution of ln(SF)**. These are based on the data in Table 1. On the right is the normal Quantile-Quantile plot for the values obtained by the bootstrap procedure.

Using the data of Table 1, we also calculated a bootstrap approximation (see Methods) to the null distribution of ln(*SF*), based on 10,000 simulated samples. This approximation does not depend on an asymptotic argument and gave *p *= 0.006. Figure 1 shows both the normal and the bootstrap approximations. The left-hand plot shows the density estimate for the bootstrap approximation and the normal density, with mean and standard deviation given by the observed ln(*SF*) and the standard error: ; the right-hand plot compares the sample quantiles of the bootstrap values of ln(*SF*) with those of a standard normal distribution, shown as a straight line. Both graphs confirm the adequacy of the normal approximation, which we also tested formally using the Kolmogorov-Smirnov test (*p *= 0.66).

The above example is of synergy between risk factors. Examples of antagonism and of protective factors are given in Additional file 2. *SF *calculations may be performed using the Excel programme in Additional file 3; an R function is available (from MCB) to compute the bootstrap approximation.

### Power

**Figure 2:**
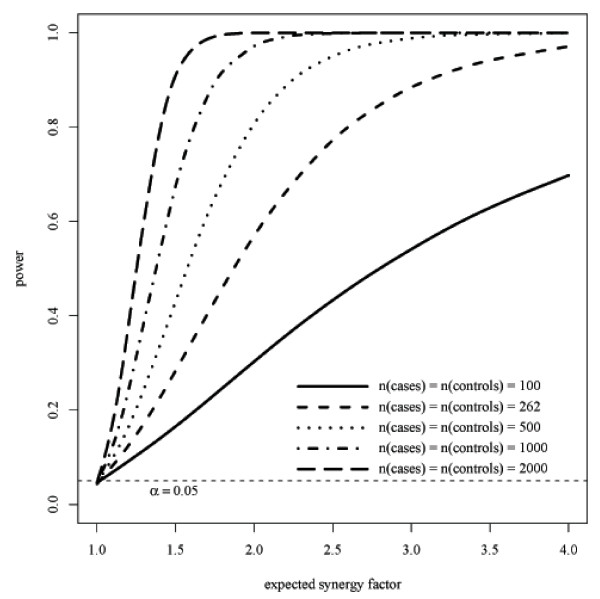
**Power curves for various sample sizes based on the control exposure frequencies in Table 1**. The example with 262 cases and 262 controls is equivalent to that of Table 1 with 252 cases and 272 controls.

Figure 2 shows power functions for different total sample sizes based on the control exposure frequencies presented in Table 1, i.e. 36% and 25% for *BACE1 *GG and *APOE*4, respectively. The total sample size of that study [13] was 524, and we also calculated the power functions corresponding to total sample sizes of 200, 1000, 2000, and 4000. When the *SF *equals 1, the power is 0.05, which is the significance level used. In this example, 488 cases and 488 controls will be needed to have 80% power to detect an *SF *of 2. Power calculations may be performed on R, using a function available from MCB.

### Meta-analyses

**Figure 3:**
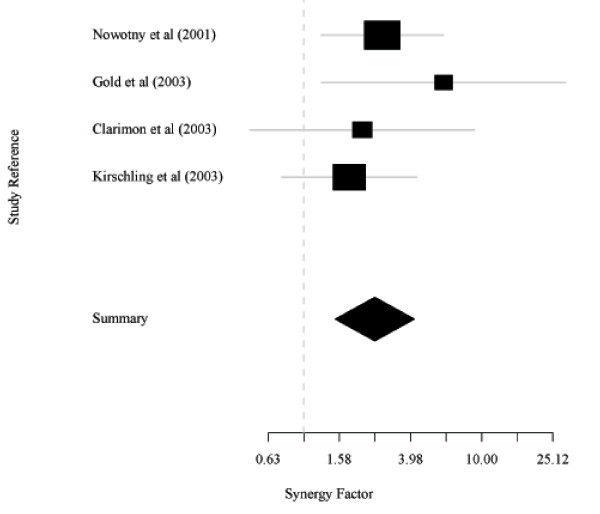
**Meta-analysis of the interaction between *BACE1 *GG and *APOE*4**. This is based on a random effects model [17].

Table 2 shows the data from 4 studies of the interaction between *APOE*4 and the *BACE1 *exon 5 GG genotype in the risk of AD [13-16]. These 4 studies are the only studies in Caucasians currently providing data to examine this interaction. Taking subjects with neither *BACE1 *GG nor *APOE*4 as reference, the pooled *SF *obtained using the random effects model [17] was 2.51, with 95% CI: 1.50–4.19 and *p *= 0.0001. The heterogeneity statistic based on three degrees of freedom was 1.88 (*p *= 0.76); the estimated random effects variance was 0. The results appear in Figure 3. Meta-analyses may be performed using the Excel programme in Additional file 4.

**Table 2:** Data for an *SF *meta-analysis of the interaction between *BACE1 *rs638405 GG and *APOE*4

**Study**	***APOE*4-positive,** ***BACE1 *GG**		***APOE*4-positive,** ***BACE1 *C+**		***APOE*4-negative,** ***BACE1 *GG**		***APOE*4-negative,** ***BACE1 *C+**	
	
	Controls	Cases	Controls	Cases	Controls	Cases	Controls	Cases
Nowotny et al 2001 [13]	19	60	48	74	80	38	125	80
Gold et al 2003 [15]	3	14	16	16	41	16	90	46
Clarimon et al 2003 [14]	4	40	10	40	21	18	52	38
Kirschling et al 2003 [16]	22	48	40	62	63	22	112	50

*APOE*4 = the ε4 allele of apolipoprotein E; *BACE1 *= the β-site APP-cleaving enzyme; GG and C+ refer to *BACE1 *exon 5 C/G (rs638405) genotypes; *APOE*4-positive and *BACE1 *C+ pool homozygotes and heterozygotes of the respective alleles

## Discussion

### The need

The real examples in Tables 1 and 2 and Table S1–S3 [Additional file 2] show the dangers of neglecting interactions. In all these examples, the effects of one or both variants were completely masked by the interacting factor. For instance, in the meta-analysis of four *BACE1 *studies (Table 2 and Figure 3), the effect of the *BACE1 *exon 5 GG was hidden in the absence of *APOE*4 [pooled *OR *= 0.8 (95% CI: 0.6–1.1; *p *= 0.17), random effects model [17]], but revealed in its presence [1.9 (1.3–2.9; 0.0015)]. Tables S1–S3 [Additional file 2] give further examples of such masking.

There is a common view that interactions, e.g. between genes (epistasis), should only be examined between risk factors that have already shown a significant main effect. But in many cases, such as most of the above, the association would be missed by the traditional single-factor approach [1-3]. Indeed, this was so in most of the examples of significant epistasis uncovered in our recent survey of sporadic AD [18]. Out of 36 such examples, 34 with *SF*s ≥ 2, the main effects of the gene variants other than *APOE*4 were generally very weak. The *OR*s were ≤ 1.2 in 20 out of 36 cases and were only significant in 5 cases. Thus, preliminary screening for main effects will miss many, possibly most cases of epistasis.

On the other hand, synergy can be too easily claimed. A common misconception is that a high combined *OR *necessarily implies synergy. A single *OR *by itself says nothing about synergy; it is the relation between the three relevant *OR*s that matters. For instance, let us assume that two risk factors are associated with *OR*s of 3 and 5 alone and of 15 when combined. Although the combined value is impressive, there is no synergy: *SF *= 15/(3 × 5) = 1. Claims of synergy are frequently published on the basis of such invalid evidence. Indeed, we have noted at least 20 claims of interactions, in the field of AD genetics alone, that were published in leading journals in recent years, but which may be clearly refuted by *SF *analysis. There is thus a need for a readily accessible method of testing such claims.

### Limitations of the SF method

We suggest that *SF *analysis, being based on logistic regression analysis, is best used for assessing binary interactions [2]. Various methods have been devised to examine higher order interactions [19,20]. However, some have only limited value for purposes of interpretation. Moreover, nearly all case-control sample-sets currently used for association studies lack the power for the proper study of higher order interactions [18]. Where a third interacting factor is suspected and a sufficiently large dataset is available, *SF *analysis may be performed twice, after stratification by the third factor, e.g. gender.

Where the relevant data are available, logistic regression analysis is the appropriate method for adjusting for covariates, while *SF *analysis should be the preferred method for stratification by covariates. Stratification can produce very small subsets, even of zero, which logistic regression analysis cannot handle. In contrast, *SF *analysis produces a realistic *p *value in each subgroup, if one adds 0.5 to each cell in any 4 × 2 table with at least one zero cell [21,22].

### Advantages of the SF method

*SF *analysis is simple to perform, through the Excel programmes in Additional files 3 and 4. It is a matter of a few minutes to perform the analysis, e.g. to check a claim of synergy in a published paper. The value of the method may be seen in the study of Combarros et al 2008 [18], in which *SF *analysis was used to examine each of the 89 studies of interactions cited in that review. The method measures both the size and significance of a binary interaction, using either primary or summarised data. Unlike logistic regression analysis, it can be applied to datasets of any size, however small, even with zero cells (above). The method can be used with all types of susceptibility factors, both risk and protective, for instance, age, gender, diet, medication or genetic polymorphisms, provided the data are dichotomised, e.g. age ± 75 years. It can be applied both to synergistic and to antagonistic interactions. Novel features include power estimation (through an R function available from MCB) and meta-analysis, an increasingly important application (through the Excel programme in Additional file 4). Neither function has been readily available before.

## Competing interests

The authors declare that they have no competing interests.

## Authors' contributions

MCB and DJL conceived the method. MCB devised the various statistical applications and worked out the necessary proofs. ADS advised on applications, e.g. in AD genetics. OC and DJL selected real examples from the AD literature and tested the method on those examples. MCB and DJL wrote the initial drafts of the manuscript and all authors read those drafts and contributed to the revisions. All authors approved the final manuscript. OC submitted the article.

## Supplementary Material

Additional file 1 **Methods**. text + 8 references. Click here for file

Additional file 2 **Examples of various types of interactions**. text + 3 tables + 3 references. Click here for file

Additional file 3 **Cortina-Borja 2009, SF calculator**. Excel programme. Click here for file

Additional file 4 **Cortina-Borja 2009, SF calculator, meta-analysis**. Excel programme with macro. Click here for file
