# Supplementary material for: The synergy factor: a statistic to measure interactions in complex diseases
Source: BMC Res Notes. 2009 Jun 15;2:105. doi: 10.1186/1756-0500-2-105 (PMC2706251; doi:10.1186/1756-0500-2-105)
Supplement: Additional file 1 — Methods. text + 8 references. [file 1756-0500-2-105-S1.doc]

**Cortina-Borja et al. The synergy factor: a statistic to measure interactions in complex diseases**

**Additional file 1**

**Methods**

***Basic analysis***

It is immediate to show that ln(*SF*), as defined above, is equivalent to the interaction term defined by two binary factors in a logistic regression model. Let *Y* be the random variable denoting the presence or absence of the outcome disease, and $x_{1},x_{2}$ denote the two exposure factors which can take the values 0 or 1. Consider the effect of one factor at a time on *Y*; let $\theta_{0}, \theta_{1}$ be the probabilities of disease for unexposed and exposed categories of any of the two risk factors. The *OR* is $\psi=\left(\theta_{1}/(1-\theta_{1})\right)/\left(\theta_{0}/(1-\theta_{0})\right)=\left(\theta_{1}(1-\theta_{0})\right)/\left(\theta_{0}(1-\theta_{1})\right)$, and its natural logarithm, i.e. the log relative risk, can be expressed as the difference between two logits: $\ln(\psi)=\ln\left(\theta_{1}/\left(1-\theta_{1}\right)\right)-\ln\left(\theta_{0}/\left(1-\theta_{0}\right)\right)=\text{logit}(\theta_{1})-\text{logit}(\theta_{0})$.

Let $\theta_{ij}$be the probability of occurrence of the disease when the two factors take the values $i,j$, where $i,j\in\{0,1\}$. Suppose that $i=0,j=0$ is the baseline category, and that $\psi_{10}, \psi_{01,} \psi_{11}$are the *OR*s with respect to this baseline for presence of $x_{1},x_{2}$ and both of them. In a logistic regression model [1]: $\text{logit(}\theta_{ij})=\ln(\theta_{ij}/(1-\theta_{ij}))=\Pr[Y=1\left|x_{1}=i,x_{2}=j]\right.=\beta_{0}+\beta_{1}x_{1}+\beta_{2}x_{2}+\beta_{12}x_{12}$. This model has four parameters $\left(\beta_{0},\beta_{1},\beta_{2},\beta_{12}\right)$ to describe four probabilities $\left(\theta_{00},\theta_{10},\theta_{01},\theta_{11}\right)$. Solving this equation explicitly for its parameters we have:

$\beta_{0}=\text{logit(}\theta_{00})$,$\beta_{1}=\text{logit(}\theta_{10}\text{)}-\beta_{0}=\ln(\psi_{10})$,$\beta_{2}=\text{logit(}\theta_{01}\text{)}-\beta_{0}=\ln(\psi_{01})$, and $\beta_{12}=\text{logit(}\theta_{11}\text{)}-\text{logit(}\theta_{00}\text{)}-\ln(\psi_{10})-\ln(\psi_{01})=\ln(\psi_{11}/(\psi_{10} \psi_{01}))$, which is ln(*SF*).

To obtain the statistical significance of *SF*, we adapt the well-known asymptotic approximation to the distribution of ln(*OR*) based on the delta method of propagation of errors [2]. It is easy to prove that under the null hypothesis, *H_0_: OR =* 1, the distribution of ln(*OR*) is asymptotically normal with mean 0 and $\mathrm{var}\left(\ln(OR)\right)=1/n_{1}+1/n_{2}+1/n_{3}+1/n_{4}$, where $n_{1},n_{2},n_{3},n_{4}$ are the frequencies of the 2 × 2 table used to compute the observed *OR*s.

Let $n_{1},n_{2},\ldots,n_{8}$ be the frequencies in a 4 × 2 table from a case control study for a binary outcome with two exposure factors $x_{1},x_{2}$:

| $x_{1}$ | $x_{2}$ | Controls | Cases |
| --- | --- | --- | --- |
| - | - | *n_1_* | *n_2_* |
| + | - | *n_3_* | *n_4_* |
| - | + | *n_5_* | *n_6_* |
| + | + | *n_7_* | *n_8_* |

Since $\ln(SF)=\ln(OR_{12})-\ln(OR_{1})-\ln(OR_{2})$, a straightforward application of the delta method [2] yields an asymptotic approximation to the standard error of ln(*SF*):

$\text{stderr}\left(\ln(SF)\right)=\sqrt{1/n_{1}+1/n_{2}+\cdots+1/n_{8}}$, and since the null value is 0, the statistic $Z=\ln(SF)/\text{stderr}\left(\ln(SF)\right)$ has, asymptotically, a standard normal distribution under the null hypothesis of no interaction. In cases where at least one cell is empty, we follow Anscombe (1956) [3] and Breslow (1981) [4], and add 0.5 to each of the 8 frequencies. A (1-*α*)% confidence interval (CI) for *SF* is $\exp\left(\ln(SF)\pm z_{1-\frac{\alpha}{2}}\text{stderr}\left(\ln(SF)\right)\right)$, where $z_{1-\frac{\alpha}{2}}$ is the corresponding quantile of a standard normal distribution.

It is possible to obtain a bootstrap approximation to the null distribution of *Z* by sampling with replacement from the frequency table conditionally on the marginals. This is a nonparametric bootstrap procedure; we have also implemented a parametric bootstrap approximation based on sampling from the Dirichlet distribution with shape parameters equal to the observed frequencies [2]. A Bayesian inferential procedure is also available [5] and we have implemented this in WinBUGS version 1.4.1.[6] In this analysis one would examine the posterior distribution of ln(*SF*), rather than construct confidence intervals based on the approximations discussed above.

***Power***

We use simulation to calculate the power for a study. We first equalize the numbers of cases and controls, adjusting the total sample size to the appropriate lower figure. Then we condition on the numbers of cases and controls and on the frequencies of the first three categories, i.e. the reference category with neither factor, that with factor A alone and that with factor B alone. We calculate the expected number of cases in the fourth category, i.e. with both factors combined, assuming appropriate *SF* values. Then we generate 10,000 samples from the corresponding binomial distributions with parameters equal to the total frequencies and proportions of cases in each category. The resulting realizations of the *SF* are used to construct power curves for the one-sided test *H_0_*: *SF* = 1 versus *H_1_*: *SF* > 1, as a function of the total sample size and of the hypothesised effects (i.e. the *SF* values chosen as appropriate).

***Meta-analyses***

It is straightforward to perform meta-analyses of synergy factors obtained from individual studies. We modify the fixed and random effects methods[7] to obtain pooled estimates (and their standard errors) of the combined *SF*. For fixed effects we use the inverse variance method: let $\xi_{i}$ denote the *i*-th estimate of ln(*SF*) out of *k* studies. The weights are obtained as the reciprocals of the variance of the estimates: $w_{i}=1/\text{stderr}(\xi_{i})^{2}$, and the inverse-variance pooled estimate is the weighted average of the *k* estimated synergy factors: $\xi_{IV}=\sum w_{i} \xi_{i}/\sum w_{i}$; its standard error is: $\text{stderr}\left(\xi_{IV}\right)=1/\sqrt{\sum w_{i}}$, and the heterogeneity statistic is $Q_{IV}=\sum w_{i}(\xi_{i}-\xi_{IV})^{2}$; under the null hypothesis it has, asymptotically, a $\chi_{(k-1)}^{2}$ distribution.

The random effects method [8] assumes that the random effects for *SF*s have a normal distribution with mean 0 and variance $\tau^{2}$ rather than a constant value. The DSL estimate of this variance is$\tau^{2}=\left(Q-(k-1)\right)/\left(\sum w_{i}-\sum w_{i}^{2}/\sum w_{i}\right)$, where *Q* is the homogeneity statistic, and it is set to 0 if *Q <* (*k -* 1). The weights used to compute $\tau^{2}$ are the inverses of the squared standard errors for each *SF* and the combined *SF* estimate is calculated as in the inverse variance method using the modified weights $w_{i}^{*}=1/\left(\text{stderr}\left(\xi_{i}\right)^{2}+\tau^{2}\right)$, so $\xi_{DSL}=\sum w_{i}^{*} \xi_{i}/\sum w_{i}^{*}$ and its standard error is $\text{stderr}(\xi_{DSL})=1/\sqrt{\sum w_{i}^{*}}$. A significance test for the null hypothesis that *SF* = 1 can be performed using $Z=\xi_{DSL}/\text{stderr}(\xi_{DSL})$, which has an asymptotic normal null distribution. The random effects variance $\tau^{2}$ (if it is larger than 0) reduces and makes more similar the weights used to compute $\xi_{DSL}$, thus producing confidence intervals wider than those obtained for $\xi_{IV}$.

1. Breslow NE, Day NJ: **Statistical methods in cancer research. Vol 1 The analysis of case-control studies**. Lyon: International Agency for Research on Cancer; 1980.

2. Tanner M: **Tools for statistical inference**. Berlin: Springer-Verlag; 1990.

3. Anscombe FJ: **On estimating binomial response relations**. *Biometrika* 1956, **43**:461-464.

4. Breslow N: **Odds ratio estimators when the data are sparse**. *Biometrika* 1981, **68**:73-84.

5. Fredette M, Angers J-F: **A new approximation of the posterior distribution of the log-odds ratio**. *Statistica Neerlandica* 2002, **56**:314-329.

6. Spiegelhalter D, Thomas A, Best N, Lunn D: **WinBUGS Version 1.4.1 User Manual, Cambridge and London**: MRC Biostatistics Unit and Imperial College; 2004.

7. Deeks JJ, Altman DG, Bradburn MJ: **Statistical methods for examining heterogeneity and combining results from several studies in meta-analysis**. In: *Systematic reviews in health care: meta-analysis in context*

Edited by Egger M, Davey Smith G, Altman DG. London: BMJ Books; 2001: 285-312.

8. DerSimonian R, Laird. N: **Meta-analysis in clinical trials**. *Control Clin Trials* 1986, **7**:177-188.
