# Supplementary material for: The synergy factor: a statistic to measure interactions in complex diseases
Source: BMC Res Notes. 2009 Jun 15;2:105. doi: 10.1186/1756-0500-2-105 (PMC2706251; doi:10.1186/1756-0500-2-105)
Supplement: Additional file 2 — Examples of various types of interactions. text + 3 tables + 3 references. [file 1756-0500-2-105-S2.doc]

**Cortina-Borja et al. The synergy factor: a statistic to measure interactions in complex diseases**

**Additional file 2**

**Examples of various types of interactions**

***Synergy between risk factors***

**Additional table 1: Odds ratios of Alzheimer’s disease, taking subjects with neither *HFE* C282Y nor *TF* C2 as reference [1]**

| *HFE* C282Y | *TF* C2 | Controls | Cases | *OR* |
| --- | --- | --- | --- | --- |
| CC  Y+  CC  Y+  Totals | -  -  +  + | 152  27  85  5  269 | 96  14  65  16  191 | Reference  0.821  1.211  5.067 |

*HFE* = the hemochromatosis gene, *TF* = transferrin, *OR* = odds ratio

Additional table 1 shows two genes of iron metabolism, the haemochromatosis gene (*HFE*) and transferrin (*TF*), with their variants, *HFE* C282Y and *TF* C2, in the risk of AD [1]. The predicted joint odds ratio (*OR*) for subjects with both *HFE* C282Y and *TF* C2 was 0.994 (= 0.821 × 1.211), compared with the observed joint *OR* of 5.067. Hence the synergy factor (*SF*) = 5.10 (= 5.067/0.994), 95% CI: 1.44, 18.0, ln(*SF*) = 1.629, stderr(ln(*SF*)) = 0.644, *Z* = 2.43, *p* = 0.011. The presence of *HFE* C282Y increased the *OR* associated with the *TF* C2 allele from 1.1 (*p* = 0.5, NS) to 4.1 (*p* = 0.004), and the presence of *TF* C2 increased the *OR* associated with the *HFE* C282Y allele from 0.8 (*p* = 0.6, NS) to 3.2 (*p* = 0.016).

***Antagonism between risk factors***

**Additional table 2: Odds ratios of Alzheimer’s disease, taking subjects with the *BLMH* 1450A allele and without *APOE*4 as reference [2]**

| *BLMH* | *APOE*4 | Controls | Cases | *OR* |
| --- | --- | --- | --- | --- |
| A+  GG  A+  GG | -  -  +  + | 222  11  77  10 | 106  20  205  26 | Reference  3.808  5.576  5.445 |

*BLMH* = bleomycin hydrolase, *APOE*4 = the ε4 allele of apolipoprotein E, A+ and GG refer to genotypes of the A1450G polymorphism of *BLMH*

Additional table 2 shows antagonism between bleomycin hydrolase (*BLMH*) 1450GG and *APOE*4 [2]. The predicted joint *OR* was 21.23 (= 3.808 × 5.576), compared with an observed joint *OR* of 5.445. Thus: *SF* = 0.26 (= 5.445/21.23), 95% CI: 0.09, 0.77, ln(*SF*) = -1.361, stderr(ln(*SF*)) = 0.558, *Z* = -2.44, *p* = 0.015. The presence of *APOE*4 reduced the *OR* associated with *BLMH* GG from 3.8 (*p* = 0.0006) to 1.0 (*p* = 1.0, NS), and the presence of *BLMH* GG reduced the *OR* associated with *APOE*4 from 5.6 (*p* < 0.0001) to 1.4 (*p* = 0.6, NS).

***Synergy between protective factors***

**Additional table 3: Odds ratios of Alzheimer’s disease, taking subjects with the *LPA* 3888P allele and without *APOE*2 as reference [3]**

| *LPA* | *APOE*2 | Controls | Cases | *OR* |
| --- | --- | --- | --- | --- |
| P+  TT  P+  TT | -  -  +  + | 96  283  18  69 | 86  256  15  14 | Reference  1.010  0.930  0.226 |

*LPA* = apolipoprotein (a), *APOE*2 = the ε2 allele of apolipoprotein E, P+ and TT refer to genotypes of the T3888P polymorphism of *LPA*

Additional table 3 shows synergy between apolipoprotein (a) (*LPA*) 3888TT and *APOE*2 [3]. The predicted joint *OR* was 0.939 (= 1.01 × 0.93), compared with an observed joint *OR* of 0.226. Thus: *SF* = 0.24 (= 0.226/0.939), 95% CI: 0.09, 0.63, ln(*SF*) = -1.422, stderr(ln(*SF*)) = 0.488, *Z* = -2.92, *p* = 0.0035. The presence of *APOE*2 lowered the *OR* associated with *LPA*3888TT from 1.0 (*p* = 1.0, NS) to 0.2 (*p* = 0.004), and the presence of *LPA*3888TT lowered the *OR* associated with *APOE*2 from 0.9 (*p* = 1.0, NS) to 0.2 (*p* < 0.0001).

**Interpretation**

For a given dataset, the choice of the order of the 4 rows in the 4 × 2 table is mainly important for interpretation. The *p* value is the same whatever the order of the rows. Also, there are only two possible *SF* values, of which one is the inverse of the other. All these *SF* calculations may be performed using the Excel programme in Additional file 3.

Every dataset offers alternative interpretations, depending on the order of the rows, since for every risk factor, there is a converse protective factor. Interpretation is not always obvious, but it is usually aided by first calculating four *OR*s, i.e. those associated with each factor in the presence or absence of the other factor. This procedure reveals one clear interpretation in each of the above examples.

1. Robson KJH, Lehmann DJ, Wimhurst VLC, Livesey KJ, Combrinck M, Merryweather-Clarke AT, Warden DR, Smith AD: **Synergy between the C2 allele of transferrin and the C282Y allele of the haemochromatosis gene (*HFE*) as risk factors for developing Alzheimer's disease**. *J Med Genet* 2004, **41**:261-265.

2. Montoya SE, Aston CE, DeKosky ST, Kamboh MI, Lazo JS, Ferrell RE: **Bleomycin hydrolase is associated with risk of sporadic Alzheimer's disease**. *Nat Genet* 1998, **18**:211-212.

3. Compton D, Wavrant DeVrièze F, Petersen RC, Tangalos E, Li L, Hardy J: **Possible association between genetic variability at the apolipoprotein(a) locus and Alzheimer's disease in apolipoprotein E2 carriers**. *Neurosci Lett* 2002, **331**:60-62.
